# Evaluation of Antibacterial Effect and Mechanical Properties of Thermoformed Clear Aligners Coated with Chitosan and Cellulose Nanocrystals 

**DOI:** 10.30476/dentjods.2025.105163.2575

**Published:** 2026-09-01

**Authors:** Seyyed Hadi Hosseini, Mohammad Motamedifar, Shabnam Ajami

**Affiliations:** 1 Postgraduate Student Orthodontic Research Center, School of Dentistry, Shiraz University of Medical Sciences, Shiraz, Iran.; 2 Dept. of Microbiology, School of Medicine, Shiraz University of Medical Sciences. Shiraz, Iran.; 3 Orthodontic Research Center, School of Dentistry, Shiraz University of Medical Sciences, Shiraz, Iran.

**Keywords:** Clear aligner, Chitosan, Cellulose nanocrystal, Antibacterial properties

## Abstract

**Background:**

Though aligners are considered more hygienic, they are linked to a higher occurrence of white spot lesions. These lesions pose clinical challenges and compromise aesthetics.

**Purpose:**

The aim of present study was the evaluation of antibacterial effect and mechanical properties of thermoformed clear aligners coated with chitosan and cellulose nanocrystals.

**Materials and Method:**

This *in vitro* study evaluated five experimental groups of aligners including (1) Passive control, (2) Active control, (3) Aligners coated with chitosan (CH), (4) Aligners coated with cellulose nanocrystals (CNCs), and (5) Aligners coated with a combination of CH/CNCs. Each group consisted of 30 samples that consisted of 10 discs for antimicrobial testing, 5 cubes for surface microhardness, and 15 cubes for the three-point bending test. Surface morphology was analyzed using scanning electron microscopy (SEM) and energy-dispersive X-ray spectroscopy (EDS). Antibacterial properties were assessed through disc diffusion and serial dilution methods against *Escherichia coli* (*E. coli*) and *Streptococcus mutans* (*S. mutans*). Mechanical properties, including surface microhardness and maximum force under bending, were evaluated using Vickers hardness and three-point bending tests. Statistical analysis was performed using ANOVA and Kruskal-Wallis tests, with *p* Value< 0.05 considered significant.

**Results:**

The CH/CNC group showing the highest microhardness (13.37±1.00 VHN) and the CNC group demonstrated the highest force resistance (62.31±7.36 MPa), though the difference was not statistically significant (*p*> 0.05). In the disc diffusion test, CH coatings showed significant antibacterial activity, especially against *E. coli* (10.8±1.09mm zone of inhibition, *p*= 0.006), and while CH/CNC showed significant antibacterial activity, especially against *S. mutans* (6.00±5.47, *p*= 0.020). CNC coatings showed no bacterial inhibitory effect (*p*> 0.05).

**Conclusion:**

The results of our study suggested that applying CNC combined with chitosan as coatings on orthodontic aligners offers a viable strategy to improve both antimicrobial and mechanical properties.

## Introduction

Clear aligner therapy utilizes transparent, thermoformed plastics that cover most or all tooth surfaces, delivering orthodontic forces to correct malocclusions [ 1 ]. Clear aligners are considered advantageous over traditional fixed appliances due to their ability to shorten treatment duration, prevent root resorption, and reduce the incidence of white spot lesions [ 2 - 4 ]. This reduction is attributed to the removability of aligners, which improves maintaining oral hygiene. However, composite attachments used to secure the aligners to specific teeth may still contribute to the formation of white spot lesions, which are difficult to remove and can cause aesthetic concerns [ 5 ]. 

To address these challenges, surface treatments and coatings have been developed to enhance the antimicrobial properties of aligner materials. Recent studies have focused on functionalizing the surface of aligners with antimicrobial agents such as essential oils, gingerol, chitosan, chitosan-based multilayer films, zinc oxide (ZnO), titanium dioxide-copper composites (TiO_2_-Cu), and cinnamaldehyde [ 1 , 6 - 11 ]. Among these, chitosan, a biopolymer derived from deacetylated chitin found in crustacean shells, has gained attention due to its biocompatibility, non-toxicity, and antimicrobial properties. Cationic nature of chitosan allows it to interact with the negatively charged cell membranes of Gram-negative bacteria, exhibiting strong antimicrobial activity against pathogens [ 12 ].

In addition to chitosan, cellulose nanocrystals (CNCs) have emerged as promising candidates for antimicrobial applications. CNCs are derived from cellulose, a biopolymer abundant in nature, and possess unique properties such as high tensile strength, low density, and large surface areas, which enhance their antimicrobial activity [ 13 ]. CNCs can interact with microorganisms due to their rigid, rod-like structure, and they have been shown to inactivate microorganisms by disrupting their cell membranes [ 14 ]. CNCs, when functionalized with antimicrobial agents such as silver (Ag) or zinc, have been shown to effectively reduce bacterial adhesion and damage bacterial membranes [ 15 - 16 ]. When CNCs combined with chitosan, the positively charged chitosan attracts Gram-negative bacteria, while CNC facilitates penetration into the bacterial wall, inducing leakage and cell lysis, which enhances antimicrobial action of chitosan [ 17 ]. While the antimicrobial efficacy of chitosan is well-established, findings regarding the antimicrobial potential of CNC remain inconsistent [ 18 - 19 ]. One research has demonstrated that CNC-coated surfaces can inactivate up to 90% of *Escherichia coli* (*E. coli*) cells, and their antimicrobial efficacy is concentration-dependent [ 20 ]. 

Considering that the main advantage of aligners is their aesthetics and the fact that many of the above mentioned materials can cause discoloration of the aligners, it was hypothesized that thermoformed aligners coated with CNCs and chitosan might provide an antimicrobial effect while maintaining the safety and mechanical properties comparing to the control group without any discoloration. The aim of this study is to evaluate the antibacterial effects and mechanical properties of thermoformed clear aligners coated with chitosan (CH) and CNCs, comparing them to uncoated control aligners. 

## Materials and Method

This *in vitro* study protocol was approved by institutional Research Ethics Committee of Shiraz University of Medical Sciences (SUMS) (IR.SUMS.DENTAL.REC. 1403.042).

In the present study, the groups were all polyethylene terephthalate glycol aligner sheets (Forplast, Roko, Poland) with an initial thickness of 1mm (before ther-moforming) and a final thickness of 0.75mm (after ther-moforming) which were evaluated in 5 groups as (1) ali-gners which no intervention was included as the passive control group, (2) aligners with surface activation as the active control group, (3) aligners coated with chitosan (CH), (4) aligner coated with CNCs, and (5) aligners coated with combination of chitosan/ CNCs (CH/ CNC). 

Each group consisted of 10-disc samples for antimicrobial testing and 5 and 15 cubic samples for microhardness and three-point bending tests.

A template was designed in the graphic design soft-ware (AutoCAD 2023, Autodesk, Inc., San Francisco, USA) to form cubes of with a size of 30×5×2mm dimension on a platform with a diameter of 10cm. The template was 3-D printed in resin (Anycubic Standard Resin, Grey, Anycubic, Shenzhen, China), and thermoforming of the aligner sheet material to the respective cubes with the dimension of 30×5×2mm were achieved using a thermoforming machine (Phrozen Sonic Mighty 8K, Phrozen, Taoyuan City, Taiwan) [ 1 ]. Aligner sheets were thermoformed on the printed blocks, and then the formed cubes were detached from the template.

### Coating of the Samples 

Five disc shaped samples and twenty thermoformed aligner material cubes in the active control group were treated with UV light for 20 minutes in a UV chamber to activate their surfaces. Following this, they were dipcoated for ten minutes with polyethyleneimine, used as a crosslinking agent. The coated cubes and discs were then placed in a hot air oven at 50°C for one hour. For the passive control group, five disc shaped samples and twenty thermoformed aligner material cubes were left untreated, with no interventions applied.

For the coated sample, 15 discs and 60 thermoformed cubes were exposed to UV light for 20 minutes to activate their surfaces. After that, they were dip-coated for ten minutes with polyethyleneimine, a crosslinking agent. For one hour, the coated cubes and discs were maintained at 50°C in a hot air oven. Following a 15-minute dip coating in the CH (20 samples), CNCs (20 samples), and CH/CNC (20 samples) solution, all the samples were baked for an hour at 50°C in a hot air oven. 

Scanning Electron Microscopy (SEM) and Energy Dispersive X-ray (EDX) analysis (Ziess, sigma 300 -HV, Germany) were used to examine the surface morphology and elemental composition of the samples.

Antibacterial activity against *Streptococcus mutans* (*S. mutans*) and *E. coli* was evaluated using the disc diffusion method. After sterilizing five discs per group with UV light, they were placed on agar plates inoculated with the test organisms. The plates were incubated at 37°C for 24 hours, and the inhibition zone was measured using a calibrated gauge and recorded in millimeters. For every sample, the zone of inhibition was performed five times and the mean of these five measurements was reported.

The antibacterial effect was further measured using a serial dilution method. The antibacterial effect was measured by growing *E. coli* in Luria broth, followed by serial dilution to achieve a culture of 1.5×10⁸ CFU/mL. The diluted culture was incubated with coated and uncoated samples for 1 and 2 hours, and bacterial colonies were counted after 24 hours. For every sample, the test was conducted five times and the mean of these five measurements was reported. The findings are shown as a CFU/mL reduction percentage.

Mechanical properties were assessed using a three- point bending test and surface microhardness testing. The maximum force (MPa) was determined using the three-point bending test (universal testing machine Zwick/Roell; z020; Germany) when each cubic specimen was deflected by 2mm at a three-point configuration with a span length of 24mm [ 21 ]. Fixing samples (15 cubic in each group) between supports and applying force perpendicular to the long axis of the samples at a rate of 0.5mm/min allowed for the execution of the three-point bending test. The mean maximum force, measured in megapascals (MPa), was reported for each group.

A Vickers microhardness tester (SCTMC MHV-1000Z; SCTMC Company, China) was used to quantify the surface microhardness at room temperature. A load of 1.96 N (200 g force) was applied for 10s (HV 0.2) to produce indentations. Five indentations with a minimum distance of 50 μ were created on each cubic sample. The mean Vickers hardness number (VHN) of each sample was then reported [ 21 ].

The data were analyzed using IBM SPSS (version 22.0). One-way ANOVA with Tukey post-hoc test was used for microhardness and bending strength comparisons. Non-parametric Kruskal-Wallis H and post hoc Mann-Whitney U tests were employed for antimicrobial results, with *p*< 0.05 considered significant. 

## Results

**Figure 1:**
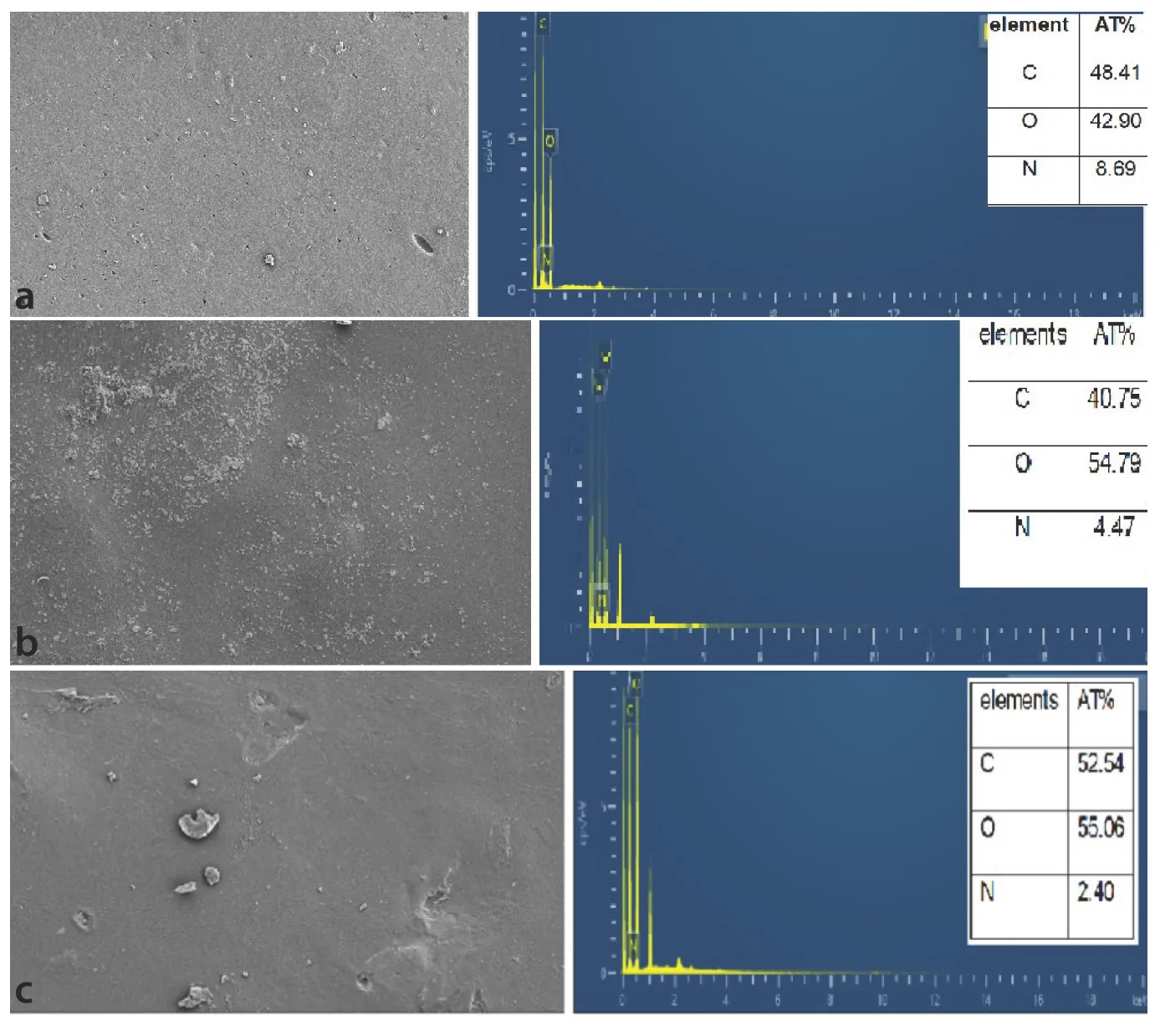
Field emission scanning electron microscopy (FESEM) with 1.00 kx magnification and elemental composition of **a:** Chitosan (CH), **b:** Cellulose nano crystals (CNC) and **c:** chitosan / Cellulose nanocrystals (CH/CNC)

 The morphology and elemental composition of the coated material were observed by SEM and images are presented in Figure 1. The CNC group showed a slightly rougher surface than that observed for pure CH and CH/CNC combination. The thickness of the coated layer was 32.8, 47.8, and 34 µm in the thickest area for CH, CNC, and the CH/CNC combination, respectively. 

The elemental composition of the aligners’ surface was carbon, oxygen, and nitrogen. Element distribution analysis showed a uniform distribution of elemental composition.

The mean±SD microhardness of different groups is shown in Table1. The highest value of micro hardness was 13.37±1.00 for the CH/CNC combination group but was not statistically significant (*p*= 0.22).

**Table 1:** Surface microhardness Vickers hardness number )VHN( (kgf/mm²)

Groups	N	Mean	S. D	*p* Value*
Passive control	5	12.86	0.32	0.22
Active control	5	12.75	0.29	
CH	5	13.00	0.17	
CNC	5	12.59	0.37	
CH/CNC	5	13.37	1.00	

*One way ANOVA test, CH: Chitosan, CNC: Cellulose nanocrystals, CH/CNC: Chitosan/Cellulose nanocrystals combination

The one-way ANOVA with multiple comparisons revealed a statistically significant difference (*p*= 0.000) among the study groups. The result of the post hoc Tukey test showed that the maximum force in the active control group was significantly (*p*< 0.05) less than in other groups (Table 2). Two by two comparison between other groups did not show a significant difference (*p*> 0.05). The highest force was demonstrated in CNC group, which was not statistically significant in comparison with other groups except when compared with active control group. 

**Table 2:** Tukey Post Hoc test results for maximum force at 2mm deflection (MPa) across study groups

Group (mean±SD)	Passive control (57.64±7.05)	Active control (50.40±4.06)	CH (57.45±8.53)	CNC (62.31±7.36)	CH/CNC (57.60±6.59)
Passive control (57.64±7.05)		*	NS	NS	NS
Active control (50.40±4.06)	*		*	***	***
CH (57.45±8.53)	NS	*		NS	NS
CNC (62.31±7.36)	NS	***	NS		NS
CH/CNC (62.31±7.36)	NS	***	NS	NS	

One way ANOVA test: * *p* Value<0.05, ** *p* <0.01, *** *p* <0.001, NS: not significant, CH: Chitosan, CNC: Cellulose nanocrystals, CH/CNC: Chitosan/ Cellulose nanocrystals combination

Table 3 summarized the mean±SD of disk diffusion assay in terms of zones of microbial growth against *E. coli* and *S. mutans*. No zone of inhibition was observed with the control groups and CNC coated samples. The multiple comparison Kruskal-Wallis H test revealed a groups for *E. coli* (*p*= 0.001) and *S. mutans* (*p*= 0.048). Table 4 showed the pairwise comparison between groups statistically significant difference among the study using Mann-Whitney U test. 

**Table 3:** Zones of microbial growth inhibition (mm) measured by disk diffusion assay against *Escherichia coli* (*S. mutans*) and *Streptococcus mutans* (*S. mutans*)

Groups	*S. mutans*	*S. mutans*
Passive control	0	0
Active control	0	0
CH	10.80±1.09	4.00±5.47
CNC	0	0
CH/CNC	6.00±5.4	6.00±5.47
*p* Value *	0.001	0.048

*Nonparametric test: Kruskal-Wallis H test, CH: Chitosan, CNC: Cellulose nanocrystals, CH/CNC: Chitosan/Cellulose nanocrystals combination

**Table 4:** Pairwise comparison of zone of inhibition between groups against *Escherichia coli* (*S. mutans*) and *Streptococcus mutans* (*S. mutans*) (mm)

Group	Passive control		Active control		CH		CNC		CH/CNC	
Bacteria	*S. mutans*	*S. mutans*	*S. mutans*	*S.mutans*	*E.coli*	*S. mutans*	*S. mutans*	*S. mutans*	*S. mutans*	*S. mutans*
Passive control			NS	NS	**	NS	NS	NS	NS	*
Active control	NS	NS			**	NS	NS	NS	NS	*
CH	**	NS	**	NS			**	NS	NS	NS
CNC	NS	NS	NS	NS	**	NS			NS	*
CH/CNC	NS	*	NS	*	NS	*	NS	NS		

*p Value< 0.05, ** *p* <0.01, *** *p*<0.001, NS: not significant, CH: Chitosan, CNC: Cellulose nanocrystals, CH/CNC: Chitosan/ Cellulose nanocrystals combination

The antimicrobial activity of CH (*p*= 0.006) coated samples against *E. coli* was significantly higher than other groups but there was not significant difference between CH and CH/CNC groups.

The antimicrobial activity of CH/CNC (*p*= 0.020) coated samples against *S. mutans* was significantly higher than other groups but there was not significant difference between CH/CNC and CH groups.

Table 5 showed the mean±SD bacterial counts for the 1-hour and 2-hour *E. coli* cultures for each group. According to the data, the CH and CH/CNC groups had lower bacterial counts at both the 1-hour and 2-hour time points compared to the other groups. The bacterial count between the 1-hour and 2-hour cultures also showed a reduction in the CH and CH/CNC groups but this difference was significant only for CH/CNC group. 

**Table 5:** Mean± SD of bacterial counts (CFU/mL) at 1-hour and 2-hour *Escherichia coli* (*S. mutans*) cultures

Groups	1 hours	2 hours	Reduction%	*p* Value*
Passive control	5*10^6^±0	4.7*10^6^±0.44	6%	0.180
Active control	5*10^6^±0	5*10^6^±0	0	1.000
CH	2.7*10^6^±0.57	1.8*10^6^±1.52	33.33%	0.276
CNC	4.8*10^6^±0.27	4.7*10^6^±0.44	2.08	0.564
CH/CNC	3.7*10^6^±0.27	2.5*10^6^±0.35	32.43%	0.042
*p* Value**	0.000	0.001		

*Wilcoxon Signed Ranks Test, ** Kruskal-Wallis H test, CH: Chitosan, CNC: Cellulose nanocrystals, CH/CNC: Chitosan/ Cellulose nanocrystals combination

Table 6 showed the pairwise comparison between groups with Mann-Whitney U test. The CH group showed a significantly lower bacterial count in the one-hour culture compared to other groups (*p*= 0.03), but this difference was not significant compared to CH/ CNC group (*p*= 0.53). In the two-hour culture, the CH and CH/CNC groups demonstrated a significant reduction in bacterial count compared to the active control group (*p*= 0.005 and 0.03), while there was no significant difference between the other groups in two-by-two comparison (*p*> 0.05). 

**Table 6:** Pairwise comparison of serial dilution method in 1h and 2h *Escherichia coli* (*S. mutans*) culture (CFU/ mL)

Group	Passive control		Active control		CH		CNC		CH/CNC	
Time	1h	2h	1h	2h	1h	2h	1h	2h	1h	2h
Passive control			NS	NS	*	NS	NS	NS	NS	NS
Active control	NS	NS			*	**	NS	NS	NS	*
CH	*	NS	*	**			*	NS	NS	NS
CNC	NS	NS	NS	NS	*	NS			NS	NS
CH/CNC	NS	NS	NS	*	NS	NS	NS	NS		

* *p* Value< 0.05, ** *p* <0.01, *** *p* <0.001, NS: not significant, CH: Chitosan, CNC: Cellulose nanocrystals, CH/CNC: Chitosan/Cellulose nanocrystals combination

## Discussion

In recent years, there has been a growing trend among the orthodontists and the patients toward the use of aligners for orthodontic treatment [ 22 - 23 ]. The application of antibacterial coatings on the surface of aligners could be beneficial, if it does not compromise aesthetics or alter their surface and mechanical properties [ 24 ].

This study used FESEM at 1000× magnification to assess the surface roughness of coated samples. The surfaces of all three coated samples were smooth, with an acceptable level of roughness, although the roughness was slightly higher on the surfaces of CNC-coated samples. Notably, the porous surface structure of the CH/ CNC coating enhanced antibacterial efficacy by increasing surface area, which facilitated stronger interactions between the coating and bacterial cells, thereby disrupting bacterial membranes more effectively.

A primary consideration in coating orthodontic devices (such as aligners) is to ensure that the antimicrobial coating does not adversely impact the mechanical and aesthetic properties of the base material [ 7 ]. Consequently, microhardness and strength of the samples were evaluated. The results of the present study indicated that the microhardness of the CH/CNC group was slightly, though not significantly, higher than that of other groups, suggesting CNC may contribute to enhancing mechanical properties which helps in reducing surface damage and wear over time, thereby maintaining the structural integrity of the appliance. This enhancement is beneficial for maintaining the structural integrity of the appliance. Similar findings by Park *et al*. [ 25 ] support this enhancement, demonstrating that CMC /CHI (carboxymethyl cellulose/ chitosan) coatings improve mechanical strength and fracture resistance. Also, the findings of Vas *et al*. [ 1 ] support this observation, as the gingerol-chitosan coating in their study increased scratch resistance, with extended coating duration further enhancing durability. In comparison, Ha *et al*. [ 9 ] observed that the TiO₂-Cu coatings increased Vickers hardness significantly, indicating enhanced wear resistance for aligners with high flexural force requirements​.

The mean maximum force in active control samples (activated without coating) was significantly lower, indicating a reduced force exerted under constant deflection. 

This effect may be due to heat treatment used for surface activation, although, clinical this observation is less relevant as surface activation without coating is not anticipated. Additionally, the maximum force in the CNC-coated group was higher than in other groups, though this difference lacked statistical significance.

The enhanced force observed in this group likely results from CNC’s inherent mechanical strength. The results also align with Yan *et al*. [ 26 ], where fluoride-coated aligners maintained mechanical stability, supporting effective treatment while offering antibacterial and remineralization benefits. In contrast, the Selenium Nanoparticles (SeNPs) in the study by Hemamalini *et al*. [ 27 ] primarily focused on antibacterial efficacy and biocompatibility rather than mechanical strength, highlighting that SeNPs may be best suited as antimicrobial agents rather than reinforcement materials.

To evaluate antimicrobial efficacy in our study, both disk diffusion and serial dilution tests were employed. Disk diffusion, a static test, assesses bacterial inhibition zones around coated materials. Since CNC lacks intrinsic antimicrobial properties, requiring active contact to be effective, the study incorporated the serial dilution test, a dynamic method, to provide a more accurate assessment of antimicrobial activity of the coated samples [ 20 ].

In particular, *E. coli* and *S. mutans* were chosen as representative bacterial models to evaluate the antibacterial efficacy of the coating due to several key considerations. *E. coli*, a Gram-negative bacterium, and *S. mutans*, a Gram-positive bacterium, were chosen to assess the effectiveness of coating across both types of bacterial cell wall structures, which may influence susceptibility to antibacterial agents [ 28 ]. *S. mutans* is a significant contributor to dental plaque formation and tooth decay, making it particularly relevant for studies targeting oral health and orthodontic applications [ 29 ]. Additionally, *E. coli* and *S. mutans* are commonly used in antibacterial research as model organisms, allowing for better comparison with other studies in the field [ 30 ].

In the disk diffusion results, only chitosan-containing compositions (CH and CH/CNC) demonstrated antimicrobial activity, with CH showing greater effectiveness against *E. coli* and CH/CNC being more effective against *S. mutans*. The stronger effect against *E. coli* likely due to electrostatic interactions between chitosan’s positive charge and the negatively charged cell wall of *E. coli*. Conversely, the CH/CNC combination showed greater activity against *S. mutans* which suggests that the combination of CNC with CH enhances its antimicrobial activity of chitosan against *S. mutans*. This outcome is consistent with the findings of Vas *et al*. [ 1 ], where a gingerol-chitosan coating significantly inhibited *S. mutans* but showed limited effects against *E. coli*. These findings support the specific targeting capabilities of chitosan-based coatings against cariogenic bacteria, such as *S. mutans*, which are relevant to oral health. However, unlike the study of Vas *et al*. [ 1 ], our study’s CH/CNC group also showed efficacy against *E. coli*, suggesting that the combination of CNC with chitosan may provide broader antibacterial coverage than gingerol alone in their study.

Preliminary laboratory tests in Vas *et al*. [ 1 ] study indicated that dip durations shorter than five minutes resulted in suboptimal crosslinking, likely due to insufficient interaction time with the crosslinking agents.

Extending the dip duration beyond 15 minutes, however, did not yield a notable increase in coating thickenss. Therefore, 5, 10, and 15 minutes were chosen as optimal intervals, with 15 minutes providing the maximum coating thickness and demonstrating the most effective antimicrobial activity against *E. coli*. Consequently, a 15-minute dip coating duration was selected for our study. 

In Vas *et al*. [ 1 ] study, the mean inhibition zone against *S. mutans* was recorded at 2.8 mm, while in our study, inhibition zones were observed at 10.8 mm for CH and 6 mm for the CH/CNC combination. These findings indicate that both CH and the CH/CNC exhibit greater antimicrobial efficacy against *S. mutans* compared to the gingerol-chitosan formulation.

Our study provides a coating solution that inherently meets aesthetic requirements, as CNC preserves transparency and does not compromise the optical clarity of aligners. Conversely, the gingerol-chitosan coating used in the study by Vas *et al*. [ 1 ] introduces pigmentation challenges that may restrict its suitability for clear aligners. Hemamalini *et al*. [ 27 ] examined the use of SeNPs to enhance the antibacterial properties of clear aligners and they showed that SeNP-coated aligners had significantly inhibited the growth of *S. mutans* and *Lactobacillus*, up to 75% of bacterial reduction. The article did not directly mention the color of the SeNPs. However, SeNPs typically exhibit a reddish or orange hue due to their nanoscale optical properties, which can sometimes affect transparency depending on concentration and coating thickness [ 31 ].

Park *et al*. [ 25 ] developed a polysaccharide-based multilayer films consisted of carboxymethyl cellulose (CMC) and chitosan (CHI) which were applied to polyethylene terephthalate glycol-modified substrates using a layer-by-layer (LbL) assembly method, while we used the dip-coating method in the present study. Dip-coating is generally faster and less labor-intensive since it only requires one immersion per coating layer. It is more efficient for single-layer or homogenous mixed-component coatings, which makes it well-suited for large-scale applications. To evaluate the antimicrobial efficacy of the incorporated polysaccharide layer, they analyzed *S. mutans* biofilm formation using electron microscopy, observing a reduction in bacterial aggregation of up to 75%. Reported bacterial presence on the superhydrophilic coated surface, with a focus on biofilm inhibition rather than direct bactericidal impact.

Unlike the direct bactericidal approach in our study, Worreth *et al*. [ 6 ] and AstasovFrauenhofer *et al*. [ 7 ] measured antimicrobial effectiveness of cinnamaldehyde-infused material and the essential oil-infused cellulose-based material through reduced biofilm formation and inhibition of metabolic activity, that showed significant antibacterial properties of those materials. Worreth *et al*. [ 6 ] showed the highest inhibition observed against *Staphylococcus epidermidis*. The *Streptococcus* strains showed a moderate response, with a 20% reduction in growth for *S. mutans* and 10% for *Streptococcus mitis*. Due to methodological differences, a direct comparison of antimicrobial outcomes could not be feasible.

In the dynamic serial dilution test, bacterial count between the one-hour and two-hours cultures was 32.43% reduction in CH/CNC group.

A significant observation is that extended contact time with the antimicrobial agents increased their efficacy (2-hour contact in culture tubes versus 1-hour). A statistically significant reduction in bacterial count was observed only in group CH/CNC, clearly indicating a synergistic antimicrobial effect of CNC when combined with chitosan. This aligns with the proposed mechanism in which positively charged chitosan attracts Gram-negative bacteria (such as *E. coli*), while CNC disrupts the bacterial cell wall, leading to leakage and cell death.

Ha *et al*. [ 9 ] investigated the TiO2-Cu composite powder coating for thermoformable orthodontic clear aligners against S.mutans. Under a 10000-fold dilution condition, the experimental group exhibited antibacterial efficiencies of 99.65 ± 0.14 % for TiO2-Cu 3 wt%, 99.35 ± 0.07 % for TiO2-Cu 5 wt%, and 99.89 ± 0.14 % for TiO2-Cu 9 wt%. These results indicate that TiO₂-Cu coatings, particularly with increased Cu content, are hig-hly effective in reducing *S. mutans*. The study suggested a 3% weight concentration as optimal for clinical use to balance aesthetic qualities with antibacterial effectiveness. While in our study, CH/CNC coatings showed strong antibacterial properties without light activation, the TiO₂-Cu films benefit from light exposure.

In contrast, Wu *et al*. [ 10 ] applied carboxybetaine methacrylate (CBMA) surface treatment to 3D- printed aligners, which demonstrated strong resistance to biofilm formation by enhancing surface hydrophilicity. CBMA’s zwitterionic properties create a hydration shell on the aligner surface, reducing bacterial adhesion without requiring antimicrobial agents​. This method differs fundamentally from the bactericidal approach of CH/ CNC [ 25 ], TiO₂-Cu [ 9 ], and SeNPs [ 27 ] coatings, as it focuses on biofilm resistance through antifouling rather than bactericidal action. In contrast to our study, which employed thermoplastic polyethylene terephthalate glycol aligners as the base material, their study [ 10 ] utilized 3D-printed (alignersGraphy, Seoul, South Korea) as the basement material. While 3D-printed materials for alig-ners offer high precision and customization, they face challenges like brittleness [ 32 ], higher costs, limited material elasticity, and potential post-processing complexities [ 33 ], may influence their overall performance compared to the traditional thermoformed materials.

Unlike the findings reported by Noronha *et al*. [ 20 ], which indicated a 90% reduction in E.coli viability on CNC-coated surfaces, our study did not reveal notable antibacterial effects for CNC-coated samples. This variation in outcomes may stem from differences in the surface material; Noronha *et al*. [ 20 ] employed a porous polyvinylidene fluoride filter, capable of holding a larger volume of CNCs, which likely intensified the interaction between CNCs and bacterial cells. Additionally, the longer 3-hour exposure time in their study may have enabled more effective and sustained CNC-bacteria contact, resulting in greater cell membrane disruption and enhanced bactericidal action.

### Clinical relevance 

Considering that one of the primary challenges in orthodontic treatment, including aligner therapy, is the development of white spot lesions and dental caries, applying cellulose nanocrystal (CNC) and chitosan coatings may be clinically advantageous. These coatings could enhance mechanical properties while simultaneously reducing cariogenic bacterial colonization, thereby lowering the risk of enamel demineralization.

## Conclusion

The results of our study suggested that applying cellulose CNC combined with chitosan as coatings on orthodontic aligners offers a viable strategy to improve both antimicrobial and mechanical properties. While CNC-coated aligner surfaces display high transparency and enhanced mechanical strength, their antibacterial effects alone are limited. However, integrating CNC with chitosan results in a composite coating that provides increased mechanical stability, effective antimicrobial protection, and satisfactory transparency. This approach addresses critical clinical challenges, such as bacterial biofilm formation and enamel demineralization during aligner therapy, thereby contributing to improved oral health outcomes in orthodontic treatment.

## References

[1] Vas NV, Jain RK, Ramachandran SK, Vas NV, Kumar Ramachandran S ( 2023). Gingerol and chitosan-based coating of thermoformed orthodontic aligners: characterization, assessment of anti-microbial activity, and scratch resistance: An in vitro study. Cureus.

[2] Ke Y, Zhu Y, Zhu M ( 2019). A comparison of treatment effectiveness between clear aligner and fixed appliance therapies. BMC Oral health.

[3] Fang X, Qi R, Liu C ( 2019). Root resorption in orthodontic treatment with clear aligners: A systematic review and meta‐analysis. Ortho Craniofac Res.

[4] Buschang PH, Chastain D, Keylor CL, Crosby D, Julien KC ( 2019). Incidence of white spot lesions among patients treated with clear aligners and traditional braces. Angle Ortho.

[5] Bisht S, Khera AK, Raghav P ( 2022). White spot lesions during orthodontic clear aligner therapy: A scoping review. J Ortho Sci.

[6] Worreth S, Bieger V, Rohr N, Astasov‐Frauenhoffer M, Töpper T, Osmani B, et al ( 2022). Cinnamaldehyde as antimicrobial in cellulose‐based dental appliances. J Appl Microbiol.

[7] Astasov-Frauenhoffer M, Göldi L, Rohr N, Worreth S, Dard E, Hünerfauth S, et al ( 2023). Antimicrobial and mechanical assessment of cellulose-based thermoformable material for invisible dental braces with natural essential oils protecting from biofilm formation. Sci Rep.

[8] Anita P, Sathyanarayana HP, Kumar K, Ramanathan K, Kailasam V ( 2023). Antimicrobial efficacy of zinc oxide nanoparticle-coated aligners on Streptococcus mutans and Candida albicans. American J Ortho Dentofac Orthop.

[9] Ha OY, Oh JM, Kim S ( 2024). Binder-Free TiO2-Cu composite powder coating for thermoformable orthodontic clear aligners. Chemical Engineering J.

[10] Wu C, Mangal U, Seo JY, Kim H, Bai N, Cha JY, et al ( 2024). Enhancing biofilm resistance and preserving optical translucency of 3D printed clear aligners through carboxybetaine-copolymer surface treatment. Dent Mater.

[11] Qin Q, Yuan W, Zhang J, Gao Y, Yu Y ( 2024). A pH-sensitive, renewable invisible orthodontic aligners coating manipulates antibacterial and in situ remineralization functions to combat enamel demineralization. Front Bioeng Biotechnol.

[12] Wieckiewicz M, W Boening  K, Grychowska N, Paradowska-Stolarz A ( 2017). Clinical application of chitosan in dental specialities. Mini Rev Med Chem.

[13] Grishkewich N, Mohammed N, Tang J, Tam KC ( 2017). Recent advances in the application of cellulose nanocrystals. Current Opinion Colloid Interface Science.

[14] Thomas P, Duolikun T, Rumjit NP, Moosavi S, Lai CW, Johan MRB, et al ( 2020). Comprehensive review on nanocellulose: Recent developments, challenges and future prospects. J Mech Behav Biomed Mater.

[15] Wang Y, Hua H, Li W, Wang R, Jiang X, Zhu M (2019). Strong antibacterial dental resin composites containing cellulose nanocrystal/zinc oxide nanohybrids. J Dent.

[16] Hemraz UD, Lam E, Sunasee R ( 2023). Recent advances in cellulose nanocrystals-based antimicrobial agents. Carbohydrate Polymers.

[17] Tyagi P, Mathew R, Opperman C, Jameel H, Gonzalez R, Lucia L, et al ( 2018). High-strength antibacterial chitosan-cellulose nanocrystal composite tissue paper. Langmuir.

[18] Muñoz-Bonilla A, Echeverria C, Sonseca Á, Arrieta MP, Fernández-García M ( 2019). Bio-based polymers with antimicrobial properties towards sustainable development. Materials.

[19] Seabra AB, Bernardes JS, Fávaro WJ, Paula AJ, Durán N ( 2018). Cellulose nanocrystals as carriers in medicine and their toxicities: A review. Carbohydr Polym.

[20] Noronha VT, Camargos CH, Jackson JC, Souza Filho  AG, Paula AJ, Rezende CA, et al ( 2021). Physical membrane-stress-mediated antimicrobial properties of cellulose nanocrystals. ACS Sustainable Chemistry Engineering.

[21] Atta I, Bourauel C, Alkabani Y, Mohamed N, Kim H, Alhotan A, et al ( 2024). Physiochemical and mechanical characterisation of orthodontic 3D printed aligner material made of shape memory polymers (4D aligner material). J Mech Behav Biomed Mater.

[22] Rosvall MD, Fields HW, Ziuchkovski J, Rosenstiel SF, Johnston WM (2009). Attractiveness, acceptability, and value of orthodontic appliances. American J Ortho Dentofac Orthop.

[23] Kau CH, Soh J, Christou T, Mangal A ( 2023). Orthodontic Aligners: Current Perspectives for the Modern Orthodontic Office. Medicina (Kaunas).

[24] Wang N, Yu J, Yan J, Hua F ( 2023). Recent advances in antibacterial coatings for orthodontic appliances. Front Bioeng Biotechnol.

[25] Park S, Kim Hh, Yang SB, Moon JH, Ahn HW, Hong J ( 2018). A polysaccharide-based antibacterial coating with improved durability for clear overlay appliances. ACS Appl Mater Interfaces.

[26] Yan J, Cao L, Luo T, Qin D, Hua F, He H ( 2023). In vitro evaluation of a novel fluoride-coated clear aligner with antibacterial and enamel remineralization abilities. Clin Oral Invest.

[27] D H, Mary P, Km S, Dhayananth X ( 2023). Evaluation of antimicrobial effect of biologically safe selenium nanoparticles-coated clear aligner: An In vitro Study. J Indian Ortho Soc.

[28] Breijyeh Z, Jubeh B, Karaman R ( 2020). Resistance of Gram-Negative Bacteria to Current Antibacterial Agents and Approaches to Resolve It. Molecules.

[29] Spatafora G, Li Y, He X, Cowan A, Tanner ACR ( 2024). The evolving microbiome of dental caries. Microorganisms.

[30] Li X, Robinson SM, Gupta A, Saha K, Jiang Z, Moyano DF, et al ( 2014). Functional gold nanoparticles as potent antimicrobial agents against multi-drug-resistant bacteria. ACS Nano.

[31] Alam MS, Javed MN, Ansari JR (2023). Metallic Nanoparticles for Health and the Environment.

[32] Jindal P, Juneja M, Siena FL, Bajaj D, Breedon P ( 2019). Mechanical and geometric properties of thermoformed and 3D printed clear dental aligners. Am J Orthod Dentofacial Orthop.

[33] Alhendi A, Khounganian R, Ali R, Syed SA, Almudhi A ( 2022). Structural conformation comparison of different clear aligner systems: An In Vitro Study. Dent J (Basel).

